# GP consultations for medically unexplained physical symptoms in parents and their children: a systematic review

**DOI:** 10.3399/bjgp13X667178

**Published:** 2013-04-29

**Authors:** Mujahed Shraim, Christian D Mallen, Kate M Dunn

**Affiliations:** Arthritis Research UK Primary Care Centre, Keele University, Keele.; Arthritis Research UK Primary Care Centre, Keele University, Keele.; Arthritis Research UK Primary Care Centre, Keele University, Keele.

**Keywords:** children, signs and symptoms, parents, primary health care, review, systematic

## Abstract

**Background:**

There is evidence of an association of medically unexplained physical symptoms (MUPS) between parents and children, but it is unclear whether this association is also present for GP consultations.

**Aim:**

To review the literature investigating the association of GP consultations for MUPS between parents and children.

**Design of study:**

Systematic review.

**Method:**

Systematic search of MEDLINE^®^, Embase, CINAHL, and PsycINFO databases from their inception to October 2012. Observational studies examining the association of GP consultations for MUPS between parents and children were included.

**Results:**

Eight studies were included in the review. Three studies found significant associations between GP consultations for multiple MUPS between parents and children. Two studies reported significant associations between irritable bowel syndrome diagnosis in parents and multiple MUPS in children. One study showed no significant associations between multiple MUPS in mothers and functional abdominal pain in children. Two studies investigated the association of non-specific low back pain in parents and children; one study showed a significant association, whereas the other study found no significant association. Formal pooling of the results was not performed owing to a high degree of study heterogeneity.

**Conclusion:**

This review provides evidence of an association between GP consultations for MUPS in parents and children, although the evidence is limited by some potential biases and study heterogeneity. GPs need to be aware of this association, which has implications for management of children presenting with MUPS. More longitudinal research focusing on all common MUPS in children, which relies on more precise sources of data, is needed to further investigate this association.

## INTRODUCTION

Non-specific physical symptoms, such as musculoskeletal pain and headache, are widespread in the community and are among the most common reasons for visiting a GP. In the UK, recent research indicates that the annual GP consultation prevalence for musculoskeletal symptoms is 25% and for headache is about 4.4%.^1^,^2^ Many physical complaints remain medically unexplained, owing to lack of obvious cause or pathological changes on physical examination and diagnostic testing. Medically unexplained physical symptoms (MUPS) are defined as physical symptoms that lead the patient to seek health care, and after clinical assessment do not seem to be explained by a clearly defined cause or diagnosis of a defined medical disease.^3^,^4^

The majority of patients presenting in primary care with MUPS improve within a few weeks,^5^ although about one-quarter of patients experience persistent or recurrent MUPS.^6^ MUPS are also common among children, and persist in a considerable proportion of children.^7^–^9^ Recurrent or persistent MUPS among children are associated with excessive utilisation of healthcare services, functional impairment, and negative impact on the quality of life of children and parents.^10^–^12^ Children with MUPS are also at greater risk of developing other MUPS and psychiatric disorders later in life.^9^,^13^,^14^

The causes of MUPS are still poorly understood, but are likely to be multifactorial. Research evidence suggests that MUPS among children may be related to a number of factors, including stressful events related to schooling and social relationships,^15^,^16^ psychopathology,^17^,^18^ childhood abuse and neglect,^19^,^20^ pubertal development,^21^ and poor parental health.^22^,^23^

Several studies have demonstrated that parental health is related to the health of the child, particularly when parents experience MUPS. Parents with MUPS and/or anxiety or depression are more likely to have children with high GP attendance rates and perceive their children to have symptoms.^22^ Children of mothers with chronic somatisation disorder (MUPS for at least 2 years) are more likely to have health problems and more GP consultations than children of mothers with explained chronic illness or mothers without chronic illness.^24^ Similarly, children of mothers with irritable bowel syndrome (IBS) have more disability days and GP consultations for gastrointestinal (GI) and non-GI symptoms than children of mothers without IBS.^23^ Some studies have focused on the associations of painful MUPS between parents and children, and reported mixed results. A few studies found no associations for any pain (musculoskeletal pain, widespread pain, and non-specific low back pain [NLBP]),^25^ functional abdominal pain (FAP),^26^ and NLBP^27^ between parents and children. Conversely, other studies found significant associations for back pain or headache,^28^–^30^ and FAP between parents and children.^31^,^32^

**How this fits in**

There is evidence of an association of medically unexplained physical symptoms (MUPS) between parents and children, but it is unclear whether this translates to similar patterns of GP consultations for MUPS between parents and children. This study found evidence of an association between GP consultations for MUPS in parents and their children. GPs need to be aware of this link, which has implications for the management and prevention of MUPS among children in primary care.

As MUPS are a significant burden in primary care, it is important to know if the association of MUPS between parents and children is also present for GP consultations. It is important to identify and better understand possible associations of GP consultation for MUPS between parents and children. It may provide valuable insights into prevention and management strategies for patients presenting with MUPS, which could improve health outcomes, quality of life, and, ultimately, reduce healthcare costs. The primary objective of this systematic review was to identify and summarise the results of observational studies, based in primary care or community settings, examining the association of GP consultations for MUPS between parents and children.

## METHOD

### Search strategy

MEDLINE^®^, Embase, CINAHL, and PsycINFO bibliographic databases were searched from their inception to October 2012. Medical Subject Heading (MeSH) and free-text terms on MUPS and primary care were used to identify papers. (The detailed search strategy is available on request from the authors.) Additionally, the reference lists of relevant papers were examined and their citations traced using the Social Science Citation Index. No restrictions were imposed on the language of publication. Local experts were contacted to identify additional relevant studies.

### Study selection

The selection included primary care and population-based observational studies that investigated the association between GP consultations for MUPS, medical diagnosis of functional somatic syndromes, or history of treated MUPS in parents and GP consultations for MUPS in children aged 1 to 17 years. It included studies in which GP consultation data for MUPS were obtained using primary care medical records, self-reported data, or both data sources. Only studies in which physical symptoms were operationally defined as MUPS or specifically referred to as functional, somatic, or non-specific were included. Studies were included regardless of the time period over which these associations had occurred.

The titles and abstracts of all studies were screened and irrelevant studies were excluded. Two reviewers assessed full-text papers to determine the eligibility of studies that appeared to meet the inclusion criteria, or when a defined decision could not be made based on the title and/or abstract alone. Any disagreements were resolved by consensus, or reconciled by a third reviewer.

### Data extraction and quality assessment

Standardised forms were used for methodological quality assessment and data extraction. The following information was extracted: study setting, design, population, number of participants and their demographic characteristics, type of MUPS, data-collection methods, and outcomes of association of GP consultations for MUPS between parents and children.

The association of GP consultations for MUPS between parents and children was defined and measured as the association between GP consultations for MUPS, history of treated MUPS, or medical diagnosis of functional somatic syndromes in parents and GP consultations for MUPS in children. The methodological quality of included studies was appraised using a methodological quality-assessment checklist for observational studies.^33^ This checklist consists of 15 items covering internal and external validity (see Appendix 1). The methodological quality for each paper was assessed independently by two reviewers. Each study was scored according to its methodological quality, using the 15-item checklist. Each item was scored positive (+) if it was satisfactorily presented, negative (−) if absent, or (na) if it was not applicable. Some items were not applicable, because of study design (no losses or dropouts in cross-sectional studies and medical record reviews). The overall methodological quality of each study was rated as ‘high’ if all or most of the items were fulfilled, ‘moderate’ if some of the items were fulfilled, and ‘low’ if few or no items were fulfilled.

## RESULTS

### Studies identified

**Figure 1:**
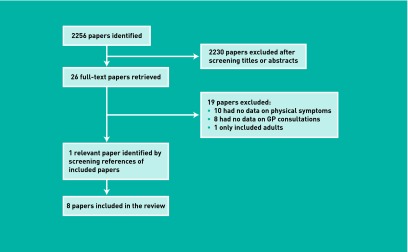
***Process of systematic search and selection of studies.***

A total of 2256 papers were identified (1106 MEDLINE, 745 Embase, 113 CINAHL, and 292 PsycINFO). Of those papers, only eight were included in the review (Figure 1).

### Quality assessment

The overall methodological qualities of included studies were high. The following items were attained by all studies: clearly defined objective, appropriate study design, representative sample, appropriate selection of outcome, appropriate measurement of outcome, standardised data collection, appropriate analysis of outcomes, and numerical description of important outcomes (Table 1).

**Table 1:** Quality assessment of included studies

**Study**	**Quality-assessment items^a^**															
	**A**	**B**	**C**	**D**	**E**	**F**	**G**	**H**	**I**	**J**	**K**	**L**	**M**	**N**	**O**	**Overall quality**
Balague *et al*, 1995^27^	+	+	+	+	−	+	+	+	+	+	na	na	+	+	+	High
Balague *et al*, 1994^29^	+	+	+	+	−	+	+	+	na	+	na	na	+	+	+	High
Campo *et al*, 2007^34^	+	+	+	+	−	+	+	+	+	na	na	na	+	+	+	High
Cardol *et al*, 2006^35^	+	+	+	+	+	+	+	+	+	na	na	na	+	+	+	High
Craig *et al*, 2002^24^	+	+	+	+	−	+	+	+	na	+	na	na	+	+	+	High
Levy *et al*, 2004^23^	+	+	+	+	−	+	+	+	+	−	na	na	+	+	+	High
Levy *et al*, 2000^36^	+	+	+	+	−	+	+	+	+	na	na	na	+	+	+	High
Little *et al*, 2001^22^	+	+	+	+	+	+	+	+	+	+	na	+	+	+	+	High

+ = satisfactorily presented.

− absent.

na = not applicable.

a See Appendix 1 for detailed description of quality-assessment items.

### Characteristics of included studies

Study characteristics are presented in Table 2. Included studies were published in English and were conducted in four different countries. Six studies were conducted in primary care and two studies identified children from schools. There were four cross-sectional surveys, three case-control studies, and one retrospective cohort study. In four studies, the parent or the child reported information on MUPS and GP consultations, and the remaining studies used either medical records alone or medical records combined with self-reported data. The mean age of children ranged between 8.5 and 14 years. The mean proportion of females was 52% (range 49% to 60%).

**Table 2:** Characteristics of included studies

**Study**	**Country**	**Setting**	**Design**	**Children’s age, years**	**Sex, % females**	**Sample size**	**Physical symptoms**	**Data source**
Balague *et al*, 1995^27^	Switzerland	School	Cross-sectional	12–17	52.5	615	NLBP	History of NLBP in parent and children was reported by children
Balague *et al*, 1994^29^	Switzerland	School	Cross-sectional	8–16	50.6	1716	NLBP	History for NLBP in parent and children was reported by children aged 13–16 years, and by parents for younger children
Campo *et al*, 2007^34^	US	Primary care	Case-control	8–15	48.5	135	FAP	History of MUPS in mothers and FAP in children was reported by mothers
Cardol *et al*, 2006^35^	The Netherlands	Primary care	Retrospective cohort	1–12	60	65 671	MUPS	Medical records review for parents and children
Craig *et al*, 2002^24^	UK	Primary care	Cross-sectional	4–8	52	151	MUPS	Medical records review for mothers; mothers reported on MUPS and GP consultations in children
Levy *et al*, 2004^23^	US	Primary care	Case-control	8–15	51	641	MUPS	Medical records review for maternal IBS and FAP and for MUPS in the child, plus self-report data on MUPS in the child by mother
Levy *et al*, 2000^36^	US	Primary care	Case-control	3–14	49	1277	GI symptoms	Medical records review for parents and children
Little *et al*, 2001^22^	UK	Primary care	Cross-sectional	<16	50	456	MUPS	History of GP consultations for MUPS in parents and children was reported by parents

FAP = functional abdominal pain. GI = gastrointestinal. IBS = irritable bowel syndrome. MUPS = medically unexplained physical symptoms. NLBP = non-specific low back pain.

### Association of GP consultations for MUPS between parents and children

Table 3 presents the associations of GP consultations for MUPS between parents and children. Six studies found significant associations between GP consultations for MUPS, history of treated NLBP or IBS in parents, and GP consultations for MUPS in children (Table 3).^22^–^24^,^27^,^29^,^34^–^36^ Four studies reported the strength of associations as adjusted odds ratios (ORs) with 95% confidence intervals (CIs), and two studies used adjusted *P*-values. Two studies did not report the strength of association, but stated that it was not significant.

**Table 3:** Associations of GP consultations for MUPS between parents and their children

**Study**	**MUPS**	**Time period**	**Summary of association**	**Factors adjusted for in multivariable analyses**	**Strength of association**			
Balague *et al*, 1995^27^	NLBP in children and parents	Lifetime	No significant association was found between parental reported history of treated NLBP and children’s lifetime history of NLBP	Child sex, age, walk time, sports activity, negative affect, positive affect, siblings’ LBP	Crude OR = 1.09, 95% CI was not reported; adjusted OR was not reported			

Balague *et al*, 1994^29^	NLBP in children and parents	Lifetime	Children of parents who had been treated for NLBP were more likely to report a history of NLBP themselves	Child age, sex, competitive sports activity, TV watched (hours/week)	Crude OR = 1.87, 95% CI = 1.42 to 2.48; adjusted OR = 2.10, 95% CI = 1.56 to 2.83			

Campo *et al*, 2007^34^	Children consulting with FAP and maternal MUPS	Lifetime	No significant association was found between child GP consultations for FAP and maternal MUPS	Maternal age, maternal psychiatric (anxiety and depressive) disorders, and family intact (child lives with biological parents)	For IBS: crude OR = 3.9, 95% CI = 1.5 to 10.3; adjusted OR = 1.8, 95% CI = 0.6 to 6.1; for migraine: crude OR = 2.4, 95% CI = 1.1 to 5.3, adjusted OR = 1.4, 95% CI = 0.6 to 3.7			

Cardol *et al*, 2006^35^	MUPS in children and parents	1 year	There was an association in GP consultation frequency for headache and abdominal pain between children and their parents compared to other families in which children consulted for physical trauma or chronic disease; association was reported as percentage of shared variance in consultation frequency between families	Child age and sex and GP practice	Percentage of variation in consultation frequency attributed to shared family factors (95% CI):			
					**Family members**	**Headache**	**Abdominal pain**	**Minor ailments**

					Mother/son	20.2 (16.4 to 24.1)	34.1 (31.0 to 37.1)	19 (18.0 to 20.0)

					Mother/daughter	48.4 (44.5 to 2.3)	34.7 (31.7 to 37.7)	23.2 (22.1 to 24.3)

					Father/son	4.7 (2.7 to 7.2)	17.1 14.4 to (19.8)	8.8 (8.0 to 9.7)

					Father/daughter	14.4 (11.1 to 18.1)	6.9 (5.1 to 8.9)	4.9 (4.3 to 5.6)

Craig *et al*, 2002^24^	MUPS in children and mothers	3 months	Children of somatising mothers had significantly more GP consultations for MUPS compared to children of control mothers	Child age and sex, child emotional or behavioural problems, mother’s exposure to adversity in her own childhood, and maternal psychiatric disorders	Adjusted *P*<0.001			

Levy *et al*, 2004^23^	GI and non-GI symptoms in children and maternal IBS diagnosis	3 years	Children of mothers with IBS had significantly more GP consultations for GI and non-GI symptoms than controls	Child age and sex, child sense of competence, child coping style, child psychological symptoms, and maternal stress, and psychological symptoms	For GI symptoms, crude *P* = 0.005 and adjusted *P* = 0.006; for non-GI symptoms, crude and adjusted *P* = 0.001			

Levy *et al*, 2000^36^	Children’s GI symptoms and parental IBS diagnosis	1 year	Children of parents with IBS had significantly more GP consultations for GI symptoms compared to control children and parents	Child age and sex, parent age and sex, parental healthcare use for non-GI disorders	Crude OR not reported, adjusted OR = 2.2, 95% CI = 1.62 to 2.98			

Little *et al*, 2001^22^	MUPS in children and parents	1 year	GP consultations for MUPS in high-attending children were significantly associated with parental GP consultations for MUPS	Child sex; parental perceived health of the child, willingness to tolerate child symptoms, health anxiety, and council house tenancy	Crude OR not reported, adjusted OR = 1.36, 95% CI = 1.10 to 1.70			

FAP = functional abdominal pain. GI = gastrointestinal. IBS = irritable bowel syndrome. LBP = lower back pain. MUPS = medically unexplained physical symptoms. NLBP = non-specific low back pain. OR = odds ratio.

One study (*n* = 456) found a significant association between self-reported GP consultations for MUPS in parents and children (OR = 1.36, 95% CI = 1.10 to 1.70).^22^ Another study (*n* = 151) showed a significant association between somatisation disorder in mothers and maternal reports of GP consultations for MUPS in children (adjusted *P*<0.001).^24^ Three studies looked at IBS; one reported significant associations between IBS in parents and recorded GP consultations for GI symptoms in 1277 children (OR = 2.2, 95% CI = 1.62 to 2.98),^36^ and another between IBS in mothers and recorded GP consultations for GI and non-GI symptoms in 641 children^23^ (adjusted *P* = 0.006 and 0.001, respectively). One study (*n* = 135) showed no significant association between history of IBS, migraine, and somatoform disorder in mothers and maternal reports of GP consultations for FAP in children (OR was reported as not significant).^34^ Two studies investigated the association of reported history of treated NLBP in parents and history of NLBP in children; one study (*n* = 1716) showed a significant association (OR = 2.10, 95% CI = 1.56 to 2.83),^29^ whereas the other study (*n* = 615) found no significant association (OR was reported as not significant).^27^ The final study (*n* = 65 671) reported the percentage of variance in similarity of recorded GP consultations among family members explained by family influence.^35^ For example, the variation in GP consultations by mothers and daughters that could be explained by family influence was 48.4% for headache and 34.7% for abdominal pain (Table 3).

Owing to the high degree of study heterogeneity between studies, pooled estimates of the strength of associations were not performed.

## DISCUSSION

### Summary

This review provides evidence that GP consultations for MUPS in parents are associated with GP consultations for MUPS in children. The review included eight papers, of which six found significant associations between GP consultations for MUPS in parents and children. Differences between studies in study designs, settings, data-collection methods, ages and numbers of included children, and types of included MUPS may partly explain the lack of association found in two studies. For example, these two studies examined the association between the lifetime prevalence of reported NLBP in children and history of treated NLBP in parents, and reported mixed findings. In the first study,^29^ schoolchildren reported information on their lifetime prevalence of NLBP as well as the history of treated NLBP in parents, whereas in the other study,^27^ both parents and children reported information on the history of their NLBP. Therefore, a possible lack of children’s knowledge of their parents’ history of treated NLBP, or recall bias, may partially explain the contradictory findings of these two studies.

The mechanisms underlying the association of GP consultations for MUPS between parents and children are not fully clear. However, there is some evidence that genetic effects,^37^,^38^ shared environmental factors,^39^,^40^ and childhood social learning of illness behaviour^24^,^36^,^41^,^42^ may explain this association. Although the majority of studies controlled for some possible confounding factors, it has been suggested that a parental decision to seek health care for their children may reflect parental health attitudes, health beliefs, and consulting behaviour, rather than the child healthcare needs.^23^,^34^,^36^ Therefore, the association of GP consultations for MUPS in parents and children may be explained by biased parental perception of symptoms in children or parental concentration on the symptoms they have themselves. For example, in one study, children with GI symptoms were interviewed independently of their mothers with IBS, and it was found that the difference between children of cases and controls was greater when the mothers reported on symptoms in children compared to children’s reports on their own symptoms.^23^ Also, the observed association of GP consultations for MUPS between parents and children may perhaps just reflect patterns of GP consultations more generally.

### Strengths and limitations

This review included only eight studies. This was despite a comprehensive search covering several electronic bibliographic databases. The citations of all included studies were searched, and no further relevant studies were identified. One relevant paper was identified through searching the references lists of included studies. The search did not address all sources of grey literature. However, local experts were contacted to identify any relevant studies, and the search was not restricted to English language publications. No studies were excluded from the review on the basis of quality assessment.

In addition to the high degree of heterogeneity among included studies, there are some limitations that should be considered when interpreting the results of this review. First, the majority of included studies relied on self-reported data, which are prone to recall bias. However, two studies examined agreement between self-reported and documented consultation for MUPS, and they showed good agreement.^22^,^24^ Second, four studies used self-reported data on the history of IBS or treated MUPS rather than patterns of GP consultations for these conditions. However, it is reasonable to suggest that those parents had to consult a medical practitioner to receive treatment and diagnosis for those conditions. Third, owing to the small number of included studies, publication bias was not assessed. Therefore, the potential for publication bias remains. Fourth, although all studies were generally of high methodological quality, only two reported a priori calculation of sample size. Finally, four studies were cross-sectional and were therefore unable to distinguish the direction of associations.

### Comparison with existing literature

This is the first systematic review to summarise the research evidence on the association of GP consultations for MUPS between parents and children. The findings from this review are in agreement with findings of other studies that specifically focused on the association of self-reported MUPS (without including GP consultations data) between parents and children, which showed mixed results.^25^,^26^,^28^,^31^,^32^,^43^ For example, two studies reported significant associations for self-reported history of FAP between parents and children,^31^,^32^ whereas this association was found to be non significant in another study.^26^

### Implications for practice

The potential impact of parental GP consultations for MUPS on the health and GP consultations of their children has implications for primary care. It is important that GPs be aware of this link, as such insights may direct the GP toward alternative management approaches. For example, cognitive behavioural therapy (CBT) targeting children’s coping responses to FAP and parents’ responses to pain in their children was associated with significant reduction in pain and MUPS severity in children in the CBT group compared to a control group.^44^ Another study showed that CBT for children with persistent MUPS and anxiety was associated with significant improvements in anxiety symptoms and reduction in pain severity and discomfort due to GI symptoms, as compared to controls.^45^

This review provides some evidence of an association between GP consultations for MUPS in parents and children. There are a limited number of studies that have investigated the association of GP consultations for MUPS between parents and children. Further longitudinal research, without relying on retrospective recall of physical symptom experience, is needed to further investigate the association between GP consultations for MUPS among parents and children. Future studies may wish to investigate this association by focusing on the whole spectrum of MUPS, including different age groups of children. Such research may provide more precise measures of the impact of parental MUPS on the health and GP consultations of their children, which has implications for the management and prevention of physical symptoms.

## Appendix

**Appendix 1.** Items used to assess the quality of observational studies

A	Clearly defined study objective
B	Appropriate design for study question
C	Inclusion and exclusion criteria clear and appropriate
D	Representative sample (and comparison)
E	Sample size calculation presented
F	Appropriate selection of outcome
G	Appropriate measurement of outcome
H	Standardised collection of data
I	Adequate length of follow-up for research question
J	Baseline participation >70% (all groups)
K	Losses and dropouts <20%
L	Adequate description of losses and completers
M	Appropriate analysis of outcomes measured
N	Numerical description of important outcomes given
O	Adjusted and unadjusted calculations provided (with confidence interval if appropriate)
